# Pulmonary Function Outcomes in Children Undergoing Diaphragmatic Plication After Phrenic Nerve Palsy Secondary to Cardiac Surgery: A 10‐Year Case Series

**DOI:** 10.1002/rcr2.70369

**Published:** 2025-10-02

**Authors:** Ehsan Aghaei Moghadam, Yousef Vojgani, Mahsa Erfanian Salim, Mohammadreza Mirzaaghayan, Behnaz Sohrabi, Hosein Ghasempour, Mohammadsadegh Talebi Kahdouei

**Affiliations:** ^1^ Fetal and Pediatric Cardiovascular Research Center Children's Medical Center Tehran Iran; ^2^ Department of Pediatric Cardiology Children's Medical Center, Tehran University of Medical Science Tehran Iran; ^3^ Pediatric Cardiac Surgeon, Department of Surgery Children's Medical Center, Tehran University of Medical Sciences Tehran Iran; ^4^ Isfahan University of Medical Sciences Isfahan Iran

**Keywords:** 6‐min walk test, diaphragmatic plication, paediatric cardiac surgery, phrenic nerve palsy, pulmonary function

## Abstract

Phrenic nerve injury is a recognised complication of congenital cardiac surgery in children, potentially leading to diaphragmatic paralysis and long‐term respiratory dysfunction. Diaphragmatic plication is performed to improve lung mechanics, but its mid‐term outcomes remain underexplored. The objective was to assess mid‐term pulmonary function in children who underwent diaphragmatic plication for phrenic nerve palsy following congenital heart surgery. This retrospective case series included five children who received left diaphragmatic plication between 2011 and 2021 at a tertiary paediatric centre. Pulmonary function was evaluated using spirometry, plethysmography, and the 6‐min walk test (6MWT), and compared to predicted normative values. At a mean follow‐up of 5 years, forced volume capacity (FVC), forced expiratory volume (FEV_1_), vital capacity (VC), and total lung capacity (TLC) were significantly lower than predicted (*p* < 0.05). FEV_1_/FVC, residual volume (RV), and forced residual capacity (FRC) were not significantly different. All patients completed the 6MWT (> 300 m), though post‐exercise oxygen saturation declined significantly (*p* = 0.011). Diaphragmatic plication leads to a restrictive pattern and exercise‐induced desaturation despite preserved walking capacity.

## Graphical abstract

This 10‐year retrospective case series evaluated mid‐term pulmonary outcomes in children who underwent diaphragmatic plication for phrenic nerve palsy following cardiac surgery. The results reveal a restrictive ventilatory pattern and consistent post‐exercise oxygen desaturation, despite preserved functional walking capacity.

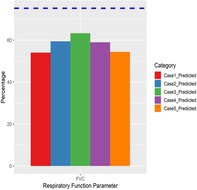

## 1 Introduction

The diaphragm is the primary muscle of ventilation, and its dysfunction is an underappreciated cause of paediatric respiratory difficulty. Diaphragmatic paralysis or severe paresis often manifests as an elevated hemidiaphragm on chest radiography and can be confirmed by fluoroscopy or ultrasound showing absent normal excursion (and paradoxical movement on sniffing) [1]. In infants and young children, the most common cause of diaphragmatic paralysis is phrenic nerve injury following cardiothoracic surgery for congenital heart disease [2, 3]. This complication can be unilateral or bilateral, with clinical presentations ranging from mild, asymptomatic breathing difficulties to severe respiratory failure [4]. Characteristic signs include difficulty weaning from mechanical ventilation, asymmetric chest expansion, recurrent atelectasis, and frequent pulmonary infections [2].

Postoperative diaphragmatic paralysis in paediatric heart surgery patients is most often caused by injury to the phrenic nerve. The phrenic nerves run along the pericardium bilaterally, making them vulnerable during cardiothoracic procedures. Mechanical trauma is a primary mechanism: surgical manoeuvres such as excessive sternal retraction, aggressive dissection near the nerve, or inadvertent traction/manipulation of the phrenic nerve can lead to neuropraxia or nerve transection [5]. For example, repairs involving the thymus or pericardium can put the nerve at risk; indeed, phrenic nerve damage has been reported during thymectomy or pericardial resection in congenital heart operations [6]. Direct injury may also occur when harvesting internal thoracic (mammary) arteries or placing sutures near the nerve's course [5, 7].

In addition to mechanical factors, thermal injury is recognised. Historically, the use of cold cardioplegia with ice slush applied to the heart and pericardium was a well‐documented cause of phrenic nerve palsy [5, 7]. Excessive cooling can cause neuropathy due to freezing of the nerve; accordingly, phrenic nerve injury was linked to myocardial ice packs in earlier cardiac surgeries [7]. Conversely, heat from electrocautery is another potential insult—if cautery is used near the phrenic nerve path, thermal spread can damage the nerve, although this is less reported than cold injury.

Ischemic injury to the phrenic nerve is a further proposed mechanism. The nerve's blood supply (via the pericardiophrenic arteries) may be compromised during surgery. Prolonged hypotension on cardiopulmonary bypass or ligation of vessels during internal mammary artery harvest could lead to ischemia of the phrenic nerve, contributing to postoperative dysfunction [5]. Excessive stretch of the nerve (or of the tissues around it) might also transiently impair perfusion to the nerve. While harder to document, ischemic neuropathy likely plays a role alongside direct trauma in some cases.

Finally, inflammatory or immune‐mediated neuropathy has been suggested as an uncommon cause of diaphragmatic paralysis after cardiac surgery. Not all cases of postoperative phrenic nerve palsy have an obvious intraoperative injury, leading researchers to consider immune mechanisms [8]. The surgical stress and cardiopulmonary bypass can trigger a systemic inflammatory response; in rare instances this may include an immune‐mediated attack on nerve fibres. There are reports of bilateral phrenic nerve paralysis resembling Guillain–Barré or neuralgic amyotrophy syndromes, where patients improved with immunotherapy (e.g., IV immunoglobulin) [8]. Such cases imply that immune‐mediated neuritis of the phrenic nerve, though rare, can occur in the post‐cardiac surgery setting.

Overall, however, the dominant pathogenesis of diaphragmatic paralysis in children remains direct iatrogenic injury to the phrenic nerve. It is “universally acknowledged” that most paediatric post‐cardiac surgery diaphragmatic paralysis stems from intraoperative nerve damage [9]. Mechanical traction, surgical manipulation or cautery, thermal injury (especially cold‐induced), and possibly localized ischemia all contribute to phrenic nerve injury during congenital heart surgeries [5, 6, 7]. The result is hemidiaphragm paralysis with paradoxical movement, which can cause respiratory compromise in infants and children. In summary, recent literature continues to support these mechanisms while also noting that an inflammatory neuropathic process may underlie a minority of cases [8]. Recognizing the cause is important, as it guides management—for instance, prompt diaphragmatic plication is advised for persistent paralysis due to nerve transection, whereas an immune‐mediated palsy might warrant immunomodulatory therapy.

Transthoracic diaphragmatic plication is a well‐established surgical treatment for phrenic nerve palsy‐induced diaphragmatic paralysis in infants and children [10]. Early plication—often within weeks of the cardiac surgery—has been shown to facilitate ventilator weaning and improve acute respiratory status [10, 11]. Previous studies have documented short‐term physiological benefits of diaphragm plication, but data on longer‐term recovery of diaphragm function and pulmonary performance are limited. Baker et al. observed that plicated hemidiaphragms can gradually regain motion over time, suggesting partial return of function [12]. However, it remains unclear to what extent paediatric patients regain normal pulmonary capacity and exercise tolerance years after plication. In particular, objective pulmonary function outcomes beyond the immediate postoperative period have not been well described in this population.

In this context, we report a 10‐year case series examining mid‐term pulmonary function in children who underwent surgical diaphragmatic plication due to phrenic nerve paralysis after congenital cardiac surgery. We describe pulmonary function test results and exercise capacity in these patients and compare their results to predicted normal values, with the aim of characterising residual respiratory impairment and guiding expectations for long‐term outcomes.

## 2 Case Series

### 2.1 Methods

This study is a retrospective single‐centre case series. It was conducted at the Children's Medical Center in Tehran, a national tertiary referral hospital for paediatric cardiology and cardiothoracic surgery. The study period spanned from January 2011 to December 2021.

We reviewed medical records of all patients diagnosed with diaphragmatic paralysis secondary to phrenic nerve injury following congenital heart surgery during the 10‐year period. Thirty‐six cases of post‐operative diaphragmatic paralysis were identified. Among these, 6 patients had died (due to either related or unrelated causes) and 9 patients did not undergo diaphragmatic plication despite the diagnosis of paralysis (thus were not treated with the intervention of interest). This left 21 patients who had received diaphragmatic plication. We applied further eligibility criteria as described below, and tracked patient outcomes via follow‐up calls and visits. After exclusions and losses to follow‐up, five patients met all criteria and were available for mid‐term evaluation of pulmonary function. Inclusion criteria were: age 6 years or older at follow‐up (to ensure reliable performance in pulmonary function testing), a documented history of hemidiaphragm plication for phrenic nerve palsy, and availability of informed consent from a parent or guardian. Patients were excluded if they were younger than 6 years, had significant cognitive or developmental impairments precluding cooperation with tests, or were unreachable for follow‐up evaluation. Applying these criteria, three patients were excluded due to age < 6. Among the remaining 18 candidates, 5 had died during the follow‐up period and 2 had neurologic developmental disabilities preventing pulmonary function testing. An additional six patients could not be contacted or did not return for evaluation. Ultimately, five patients (out of the original 36) fulfilled all criteria and completed the study assessments.

- Timing of Measurements: Baseline vitals (HR and SpO_2_) should be recorded after ~10 min of rest before the test. Immediately upon completing the 6‐min walk, HR and SpO_2_ are measured (within seconds) while the child is standing still. This captures the peak exercise heart rate and the lowest oxygen saturation (which may occur at end exercise or just after stopping). Guidelines emphasise that these post‐walk values be taken *immediately* at test end (and not delayed by several minutes) to reflect the acute exercise response. If a second 6MWT is performed (for learning effect), the child must rest until HR and SpO_2_ return to baseline before repeating.
- Device Type for HR and SpO_2_: A portable pulse oximeter is the recommended device for monitoring SpO_2_ and heart rate during paediatric 6MWTs. Continuous pulse oximetry throughout the walk is now advised so that the nadir SpO_2_ (lowest saturation) can be captured, as it is an important marker of disease severity. The pulse oximeter probe (typically on a finger or earlobe) should be lightweight, battery‐powered, and secured (e.g., in a fanny pack) so the child does not have to hold it and so that it does not impede walking. Routine ECG monitoring is not required for a standard 6MWT; heart rate can be reliably obtained from the pulse oximeter. Continuous ECG or telemetric monitors are only used in special cases (e.g., patients at risk for arrhythmia), as they are not part of the standard protocol.
- Averaging Period for Readings: While official paediatric 6MWT guidelines do not specify an exact averaging interval for pulse oximetry, they stress the importance of obtaining a *stable* reading. The oximeter's settings should follow manufacturer guidelines to balance responsiveness with accuracy. In practice, using a short averaging time (e.g., 3–4 s) on modern pulse oximeters is recommended to detect transient desaturations. The key is to document the lowest stable SpO_2_ value observed (rather than a fleeting artefact) and the corresponding end‐test heart rate. Typically, the SpO_2_ and HR displayed at the end of 6 min (or immediately after stopping, once the child stands still) are recorded, which effectively averages the last few seconds of exercise.
- Handling Motion Artefacts and Variability: Motion artefact is a known challenge during ambulation. Earlier ATS guidelines noted that many pulse oximeters can give erroneous readings during walking due to motion. To mitigate this, the probe should be well‐secured and the child instructed to relax the hand. The technician should avoid walking next to or talking to the child except for standardised encouragement, as hovering can alter the child's pace and also isn't needed for monitoring. Instead, the proctor walks slightly behind, observing the oximeter discreetly so as not to ‘pace’ the child. If continuous readings are unstable due to artefact, priority is given to the value obtained immediately once the child stops (when movement ceases and the signal stabilises). Following manufacturer instructions (e.g., ensuring good sensor contact and perfusion) helps minimise data dropouts. In summary, stable values should be recorded—if the immediate post‐walk SpO_2_ reading is erratic, one may use the value a few seconds after stopping, once the signal is reliable. The lowest SpO_2_ during the test (if observed on a well‐functioning probe) should be noted, with an annotation if poor signal quality was suspected. Using newer pulse oximeters with motion‐correction algorithms can further improve accuracy. Overall, the latest standards encourage continuous monitoring to capture true physiological responses while advising vigilance against artifactual drops or spikes in the readings.

All patients had previously undergone surgical diaphragmatic plication on the affected (paralysed) hemidiaphragm within the initial hospitalisation period after their cardiac surgery. In all five cases, the paralysis involved the left hemidiaphragm, and plication was performed via an open transthoracic approach soon after the diagnosis of diaphragmatic paralysis was confirmed. No perioperative complications of the plication procedures were documented in the medical records, and each patient was successfully weaned off mechanical ventilation and discharged in stable condition following plication. All included patients developed diaphragmatic paralysis shortly after their initial cardiac surgery, confirmed by fluoroscopy during the same hospitalisation. Surgical diaphragmatic plication was performed soon after diagnosis, generally within 1–2 weeks, while the patients were still in the postoperative care period.

Mid‐term follow‐up assessments were performed for each of the five patients in a standard fashion. All evaluations took place in the same paediatric pulmonary laboratory using calibrated equipment and were conducted by experienced respiratory technicians. Pulmonary function tests (PFTs) included spirometry, whole‐body plethysmography, and a 6‐min walk test (6MWT). Spirometric measurements included vital capacity (VC), forced vital capacity (FVC), forced expiratory volume in 1 s (FEV_1_), FEV_1_/FVC ratio, and forced expiratory flow at 25%–75% of vital capacity (FEF_50_ or FEF_25–75_). Lung volumes measured by body plethysmography included total lung capacity (TLC), functional residual capacity (FRC), and residual volume (RV). The 6MWT was conducted according to American Thoracic Society guidelines: each child was instructed to walk as far as possible in 6 min along a marked corridor, with standard encouragement. Distance walked (in meters) was recorded. Peripheral oxygen saturation (SpO_2_) and heart rate were measured by pulse oximetry at rest before the test and immediately upon completion of the 6‐min walk [13]. All five patients completed the tests successfully.

Predicted normal values for spirometry and lung volumes were calculated for each child based on age, sex, height, and ethnicity, using internationally recognised paediatric reference standards [13, 14]. For spirometric indices (VC, FEV_1_, FVC), at least 75% of the predicted value was considered within the normal range for that age [14]. For FEV_1_/FVC, a normal value was defined as ≥ 85% [14], and for mid‐expiratory flow (FEF_25–75_), ≥ 65% of predicted was considered normal [14]. Predicted normal ranges for lung volumes were defined as follows: TLC 80%–130% of predicted, FRC 70%–130%, and RV 65%–130% of predicted [13]. Each patient's measured values were compared against these reference thresholds to determine whether a restrictive or obstructive ventilatory defect was present.

For group‐level analysis, we compared the patients' pulmonary function results to their predicted values using paired‐sample *t*‐tests (treating each patient's measured vs. predicted value for a given parameter as a pair). Given the small sample size, these analyses were exploratory in nature. A two‐tailed *p* < 0.05 was considered statistically significant. Statistical analysis was performed using R software (R Foundation for Statistical Computing, Vienna, Austria; version 4.2.0). In addition, descriptive statistics are used to summarize the cohort where appropriate. Results are presented in both narrative form and in tabular form (Table 1).

**TABLE 1:** Patient demographics, cardiac diagnoses, hemidiaphragm paralysis side, and key pulmonary function and exercise results at follow‐up (percentage of predicted value shown where applicable).

Case	Age at follow‐up (years)	Cardiac diagnosis	Side of diaphragm paralysis	VC (%pred)	FEV_1_ (%pred)	FVC (%pred)	TLC (%pred)	6MWT distance (m)	SpO_2_ before/after (%)	PFT pattern
1	7	DORV, PA, ASD, VSD	Left	↓ (63%)	↓ (61%)	↓ (65%)	Normal (92%)	450	85/78	Mixed (restrictive/obstructive)
2	6	SV, PS, PDA	Left	Normal	Normal	Normal	Normal	315	92/79	Normal
3	7	TGA, PA, Situs inversus	Left	Normal	Normal	Normal	Normal	450	98/93	Normal
4	6	TOF	Left	↓ (69%)	↓ (66%)	↓ (68%)	↓ (76%)	337.5	97/91	Restrictive
5	7	TOF, DORV, PS, VSD	Left	Normal	Normal	Normal	Normal	395	93/89	Normal

Abbreviations: 6MWT = 6‐min walk test; PFT = pulmonary function test; R/O = restrictive/obstructive; SpO_2_ = oxygen saturation.

### 2.2 Results

#### 2.2.1 Patient Characteristics

Five patients (three males and two females) met inclusion criteria and underwent pulmonary follow‐up testing. All had a history of congenital heart disease requiring surgery in infancy, complicated by left phrenic nerve palsy and diaphragmatic paralysis. Each patient underwent left hemidiaphragm plication in the neonatal period or early infancy as described above. At the time of pulmonary evaluation, the patients were 6–7 years old (median age 7). Table 1 summarises the key cardiac diagnoses, age at follow‐up, side of diaphragm paralysis, and the pulmonary function and exercise results for each case.

#### 2.2.2 Case 1

**FIGURE 1:**
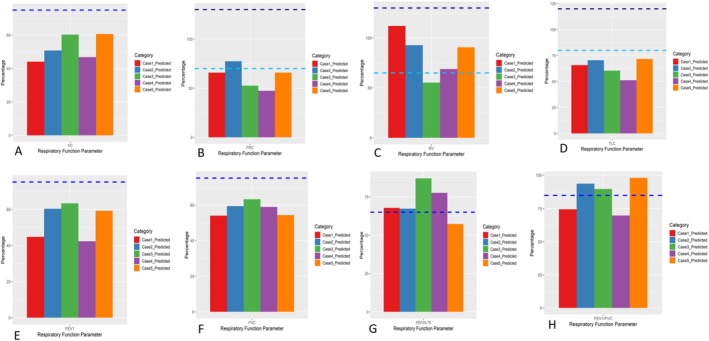
(A) Comparison of patients' VCs with the normal percentage of predicted = 75% (dashed horizontal line). (B) Comparison of patients' FRCs with the lower limit normal percentage of predicted = 70% (light blue dashed horizontal line) and upper limit of normal = 130%. (C) Comparison of patients' RVs with the lower limit normal percentage of predicted = 65% (light blue dashed horizontal line) and upper limit of normal = 130%. (D) Comparison of patients' TLCs with the lower limit normal percentage of predicted = 80% (light blue dashed horizontal line) and upper limit of normal = 130%. (E) Comparison of patients' FEV1s with the normal percentage of predicted = 75% (dashed horizontal line). (F) Comparison of patients' FVCs with the normal percentage of predicted = 75% (dashed horizontal line). (G) Comparison of patients' FEFs 25–75 with the normal percentage of predicted = 65% (dashed horizontal line). (H) Comparison of patients' FEV1s/FVCs with the normal percentage of predicted = 85% (dashed horizontal line).

A 7‐year‐old girl with complex congenital heart defects (dextrocardia, double outlet right ventricle with large ventricular septal defect, large atrial septal defect, and pulmonary atresia) underwent multiple cardiac interventions in infancy, including open‐heart surgery and catheter‐based stent placement. She re‐presented at 8 months old with cyanosis and respiratory distress. Fluoroscopy at that time revealed left hemidiaphragm paralysis consistent with phrenic nerve palsy (a complication of her prior cardiac surgery). She underwent left diaphragmatic plication and was discharged a few days later in stable condition. Approximately 5 years after plication (at age 7), her follow‐up 6MWT distance was 450 m in 6 min. Heart rate was 115 beats/min at rest and 110 beats/min after exercise, and oxygen saturation declined from 85% at baseline to 78% post‐walk. Pulmonary function testing showed significantly reduced volumes compared to predicted normals (overall *p* = 0.007 for difference from predicted). In particular, her VC, FEV_1_, and FVC were all only ~60%–65% of predicted, although TLC and RV were within normal range (Table 1, Figure 1). Despite the anticipated restrictive pattern due to the plicated diaphragm, a mild obstructive component was also evident in her results (her FEV_1_/FVC was slightly below normal). Notably, she had a personal and family history of asthma; her parents reported that she experiences wheezing and prolonged cough during upper respiratory infections. This suggests that her pulmonary function pattern may represent a combination of restrictive impairment from the diaphragm paralysis and a superimposed mild obstructive airway disease.

#### 2.2.3 Case 2

A male infant was diagnosed at birth with severe cyanotic congenital heart disease, including single‐ventricle physiology with severe pulmonary stenosis (intact ventricular septum) and a large patent ductus arteriosus. He underwent neonatal cardiac surgery to address these defects. In the postoperative period, while in the neonatal intensive care unit, he developed notable respiratory distress with chest wall retractions. Fluoroscopic examination demonstrated left diaphragmatic paralysis. He underwent left hemidiaphragm plication approximately 1 week later and was subsequently discharged in stable condition. At 6 years of age, his mid‐term evaluation showed no significant pulmonary function deficits. His spirometry and lung volumes were essentially within normal limits relative to predicted values (*p* = 0.11 for the difference) (Table 1). At follow‐up, he walked 315 m during the 6MWT. His heart rate was 90 beats/min pre‐exercise and 80 beats/min after the walk. Oxygen saturation dropped from 92% at rest to 79% after exercise. Despite this desaturation, his exercise performance and pulmonary function results were largely unremarkable, indicating a good functional recovery of respiratory capacity.

#### 2.2.4 Case 3

A 7‐year‐old girl with situs inversus (mirror‐image organ arrangement) was evaluated for central cyanosis at 6 months of age. Echocardiography revealed dextrocardia with transposition of the great arteries (TGA), pulmonary atresia, and right pulmonary artery stenosis. She underwent corrective cardiac surgery in infancy. About 5 years later, during routine follow‐up at age 5, she exhibited mild respiratory symptoms, including exertional dyspnea and diminished breath sounds on the left side. Chest X‐ray showed elevation of the left hemidiaphragm (eventration), and fluoroscopy confirmed left diaphragm paralysis. She underwent left diaphragmatic plication. At 7 years old, her PFT results were within normal ranges (no significant difference from predicted values, *p* = 0.16) (Table 1). She walked 450 m in the 6MWT; heart rate was 104 beats/min pre‐test and 106 beats/min post‐test. Her oxygen saturation was 98% at rest and 93% after walking. Overall, her mid‐term pulmonary function and exercise tolerance were normal, reflecting an excellent outcome after plication.

#### 2.2.5 Case 4

A 6‐year‐old boy with tetralogy of Fallot (TOF) underwent surgical repair in infancy. His postoperative course was complicated by prolonged ventilator dependence for about 1 month. Difficulty in weaning and persistent respiratory distress (tachypnea with subcostal retractions) prompted further evaluation. Fluoroscopy revealed left diaphragmatic paralysis. He subsequently underwent left hemidiaphragm plication. After plication, he was successfully weaned from mechanical ventilation and discharged with an oxygen saturation of 97% in room air and no significant respiratory symptoms. At 6 years of age, this patient's pulmonary function demonstrated a restrictive deficit. His VC, FEV_1_, FVC, and TLC were all markedly reduced (approximately 66%–76% of predicted values; see Table 1, Figure 1), reflecting significantly lower lung volumes than expected (*p* = 0.013 for overall difference from predicted). Consistent with a pure restrictive pattern, his FEV_1_/FVC ratio remained normal. On the 6MWT, he walked 338 m in 6 min. Heart rate was 109 beats/min before exercise and 103 beats/min after. Oxygen saturation dropped from 97% at rest to 91% after the walk. These findings indicate that, despite successful surgical plication and resolution of acute symptoms, this child had persistent reductions in lung volumes in the mid‐term, consistent with lasting consequences of the early diaphragm paralysis.

#### 2.2.6 Case 5

A male neonate with multiple complex cardiac anomalies (TOF with double outlet right ventricle, pulmonary stenosis, and a large VSD) was noted to have a loud holosystolic heart murmur and perioral cyanosis shortly after birth. He underwent open‐heart surgery in the first week of life to repair the cardiac defects. Within days of surgery, he developed cyanosis of the lips and distal extremities and difficulty breathing during feeding. Fluoroscopy confirmed left hemidiaphragm paralysis, and a left diaphragmatic plication was performed to resolve the respiratory compromise. By the time of follow‐up at 7 years old, this patient's PFT results were within normal limits relative to predictions (*p* = 0.055, indicating no statistically significant deficit). In the 6MWT, he walked 395 m. His heart rate was 96 beats/min pre‐test and 91 beats/min post‐test, and his oxygen saturation changed from 93% at rest to 89% after exercise. Thus, like Cases 2 and 3, Case 5 demonstrated generally normal spirometry and lung volumes at mid‐term, along with good exercise performance, apart from a mild desaturation during exertion (Table 1, Figure 1).

## 3 Discussion

In this case series of five paediatric patients, we found that children who had diaphragmatic plication for phrenic nerve paralysis after cardiac surgery exhibit notable long‐term changes in pulmonary function. All patients demonstrated a pattern consistent with restrictive lung disease on formal PFTs, characterised by reductions in lung volumes (VC, FEV_1_, FVC, TLC) relative to predicted values. This is an expected consequence of diaphragmatic paralysis, as the elevated hemidiaphragm and loss of diaphragmatic contractility limit lung expansion [4, 15]. Our findings align with prior observations that phrenic nerve injury in infants leads to a restrictive respiratory physiology when tested [4]. Notably, in our cohort the group differences for FVC, FEV_1_, VC, and TLC versus normal reference values were all statistically significant, reinforcing the presence of a persistent restrictive deficit even several years after the plication procedure.

Despite the reduction in volumes, indices of expiratory flow and air trapping were largely within normal ranges for our patients. For instance, the FEV_1_/FVC ratios remained normal in four of five cases, and group analysis showed no significant difference in FEV_1_/FVC between patients and predicted norms. Forced Mid‐expiratory flows (FEF_25–75_) were also not significantly different from expected values, and almost all patients had RV and FRC within normal limits (Table 1). These findings indicate that, in the absence of other pulmonary conditions, diaphragmatic paralysis primarily causes a restrictive impairment without intrinsic airway obstruction. One patient (Case 1) did have a mixed pattern (low FEV_1_/FVC along with low volumes), which we attribute to coexistent mild asthma; this underscores that individual variations can occur if other respiratory comorbidities are present. Overall, our results suggest that the long‐term pulmonary impact of infant diaphragmatic paralysis (after surgical plication) is predominantly a reduction in lung volumes rather than airflow obstruction.

Phrenic nerve paralysis after paediatric cardiac surgery is a well‐documented complication, with incidence reported in up to a few percent of cases in older series [16]. Early spontaneous recovery of phrenic nerve function is rare, especially if no improvement is seen within the first month post‐injury [17]. Thus, surgical plication of the diaphragm is generally recommended if diaphragmatic paralysis persists beyond a few weeks and the patient has significant respiratory compromise [18]. In all our cases, plication was performed within weeks of the initial injury, which is consistent with recommended practice to optimise short‐term outcomes [10, 18]. Plication effectively tethers the affected hemidiaphragm in a lower (flattened) position, preventing paradoxical movement and allowing better lung expansion. Prior studies have shown that this leads to immediate improvements in lung function and oxygenation, often evident intraoperatively or in the early postoperative period [19]. Our findings extend these observations by showing that, several years later, children who underwent plication can achieve near‐normal exercise capacity, though their static pulmonary function measures remain depressed compared to healthy peers. This persistent reduction in lung volumes could reflect lasting changes in respiratory mechanics due to the initial paralysis and scarring of the diaphragm, or perhaps suboptimal catch‐up growth of the ipsilateral lung.

Importantly, exercise testing in our series revealed that all children could perform a 6MWT to a distance considered normal for their age (generally, healthy children > 6 years are expected to walk at least ~300 m in 6 min) [13]. However, every patient in our series experienced a drop in oxygen saturation during exercise, from an average baseline SpO_2_ in the mid‐1990s to the high 1970s or 1980s after the walk. Such exercise‐induced desaturation is not typical in healthy children [13] and suggests a limitation in pulmonary reserve or gas exchange efficiency among these patients. The mechanism may relate to a reduced ventilatory capacity (due to the smaller effective lung volume) leading to relative hypoventilation or atelectasis during exertion, or ventilation‐perfusion mismatch in the previously affected lung. Nevertheless, the fact that all patients maintained exercise endurance within normal limits indicates that, functionally, they are able to compensate for their restrictive impairment in everyday activities up to a point. This finding is clinically reassuring, as it implies that childhood diaphragm plication, despite leaving some pulmonary function deficits, allows good functional status in mid‐term follow‐up.

Adult case series have reported outcomes of diaphragmatic plication in patients with phrenic nerve paralysis, with findings broadly consistent with our paediatric results. Simansky et al. compared paediatric and adult cohorts and showed that both groups exhibited restrictive ventilatory defects after plication, but symptomatic relief was generally greater in children. In adults, lung volume improvements following plication tend to be modest and sometimes decline over time, while children may demonstrate partial recovery of function due to lung growth and compensatory mechanisms [11]. Other adult studies have also documented reduced static lung volumes and persistent restrictive physiology after plication, though exercise tolerance and dyspnea scores often improve [7, 15]. Compared to these adult cohorts, our paediatric patients demonstrated a similar restrictive pattern but retained good functional walking capacity, which may reflect the greater adaptability of the developing paediatric respiratory system.

Our case series has several limitations. First, the sample size is small, reflecting the rarity of this complication and the challenges in long‐term follow‐up. We initially identified 36 children with phrenic nerve palsy after cardiac surgery over a decade; however, many could not be included due to death or loss to follow‐up. The five cases presented may therefore represent a subset of patients healthy enough to return for testing, which could bias the results toward better outcomes. Second, as a retrospective analysis without pre‐plication PFT data (infants are incapable of performing such tests), we cannot quantify the improvement attributable to plication in each case or track trajectory of lung function development. Our comparisons relied on normative predicted values rather than each patient's own baseline, which is an inherent limitation when studying an intervention in infancy. Third, we did not have a control group of non‐plicated patients or healthy peers; our statistical comparisons were to reference standards. Despite these limitations, a strength of this study is the detailed physiological assessment of each child, including comprehensive lung volume measurements and exercise testing, which provide insight into the practical outcomes of early diaphragmatic plication. All evaluations were performed in a uniform manner at a single centre, lending internal consistency to the data.

This report highlights that moderate restrictive lung deficits can persist in the mid‐term years after infant diaphragmatic plication, even though clinical recovery may appear complete. Larger, multicentre studies or patient registries would be valuable to confirm these findings and to determine the variability of outcomes across different centres and surgical techniques. Future research could also explore whether any interventions (such as respiratory muscle training, pulmonary rehabilitation, or newer surgical approaches like minimally invasive plication) might further improve long‐term pulmonary function in these children. Finally, long‐term follow‐up into adolescence and adulthood would be informative to see if lung function deficits persist, worsen, or improve (e.g., as growth allows more lung development or as collateral innervation of the diaphragm occurs). Understanding these outcomes will help clinicians provide prognostic information to families and devise optimal management strategies for patients with this challenging complication.

In summary, children who underwent diaphragmatic plication for phrenic nerve palsy following congenital heart surgery showed significantly reduced static pulmonary function indices at mid‐term follow‐up, consistent with a restrictive ventilatory pattern. Despite these impairments, their exercise capacity (6‐min walk distance) was relatively well‐preserved, though exercise‐induced oxygen desaturation was a consistent abnormal finding. These results underscore that diaphragmatic paralysis in infancy can have measurable longer‐term impacts on lung function, even when overt clinical symptoms have resolved. Early surgical plication remains a crucial intervention to stabilise the condition and appears to allow good functional recovery. However, continued surveillance of respiratory health in such patients is warranted. This case series emphasises the need for further research, ideally in larger cohorts, to better characterise long‐term pulmonary outcomes and to guide postoperative care and counselling in children with phrenic nerve injury and diaphragm plication.

## Author Contributions

**Ehsan Aghaei Moghadam:** conception or design of the work. **Yousef Vojgani:** the acquisition, analysis or interpretation of data for the work. **Mahsa Erfanian Salim, Mohammadreza Mirzaaghayan** and **Behnaz Sohrabi:** drafting the work or reviewing it critically for important intellectual content. **Mohammadsadegh Talebi Kahdouei and Hosein Ghasempour:** final approval of the paper.

## Ethics Statement

The study was approved by the Institutional Ethics Committee of Children's Medical Center, Tehran University of Medical Sciences. Approval number: IR.TUMS.CHMC.REC.1400.031.

## Consent

The authors declare that written informed consent was obtained from the parents or legal guardians of all patients for the publication of this manuscript and accompanying data, and attest that the form used to obtain consent complies with the Journal requirements as outlined in the author guidelines.

## Conflicts of Interest

The authors declare no conflicts of interest.

## Data Availability

The data that support the findings of this study are available from the corresponding author upon reasonable request.

## References

[1] L. K. Nason , C. M. Walker , M. F. McNeeley , W. Burivong , C. L. Fligner , and J. D. Godwin , “Imaging of the Diaphragm: Anatomy and Function,” Radiographics 32, no. 2 (2012): E51–E70. 22411950 10.1148/rg.322115127

[2] N. Gerard‐Castaing , T. Perrin , C. Ohlmann , et al., “Diaphragmatic Paralysis in Young Children: A Literature Review,” Pediatric Pulmonology 54, no. 9 (2019): 1367–1373. 31211516 10.1002/ppul.24383

[3] P. Akbariasbagh , M. R. Mirzaghayan , N. Akbariasbagh , M. Shariat , and B. Ebrahim , “Risk Factors for Post‐Cardiac Surgery Diaphragmatic Paralysis in Children With Congenital Heart Disease,” Journal of Tehran Heart Center 10, no. 3 (2015): 134–139. 26697086 PMC4685369

[4] A. Baskaralingam , L. Nicod , and R. Manzoni , “Diaphragmatic Paralysis and Paresis : Review,” Revue Médicale Suisse 16, no. 705 (2020): 1646–1651. 32914596

[5] J. Whiteley , M. Shoeib , and R. Bilancia , “Iatrogenic Phrenic Nerve Palsy,” Shanghai Chest 5 (2021): 5.

[6] E. Arslanoğlu , S. Bakhshaliyev , K. A. Kara , et al., “Congenital Cardiac Surgery and Diaphragmatic Paralysis: Efficacy of Diaphragm Plication,” Cardiothoracic Surgeon 32, no. 1 (2024): 5.

[7] L. C. H. F. Tripp and L. C. J. R. Bolton , “Phrenic Nerve Injury Following Cardiac Surgery: A Review,” Journal of Cardiac Surgery 13, no. 3 (1998): 218–223. 10193993 10.1111/j.1540-8191.1998.tb01265.x

[8] P. Ripellino , M. Pons , M. G. A. Izzo , and C. Gobbi , “Bilateral Phrenic Neuropathy Responsive to Intravenous Immunoglobulin Treatment,” Clinical and Translational Neuroscience 3, no. 2 (2019): 2514183X19891606.

[9] M.‐S. Hwang , J.‐J. Chu , and W.‐J. Su , “Diaphragmatic Paralysis Caused by Malposition of Chest Tube Placement After Pediatric Cardiac Surgery,” International Journal of Cardiology 99, no. 1 (2005): 129–131. 15721511 10.1016/j.ijcard.2003.10.060

[10] R. Shoemaker , G. Palmer , J. W. Brown , and H. King , “Aggressive Treatment of Acquired Phrenic Nerve Paralysis in Infants and Small Children,” Annals of Thoracic Surgery 32, no. 3 (1981): 250–259. 7283517 10.1016/s0003-4975(10)61047-8

[11] D. A. Simansky , M. Paley , Y. Refaely , and A. Yellin , “Diaphragm Plication Following Phrenic Nerve Injury: A Comparison of Paediatric and Adult Patients,” Thorax 57, no. 7 (2002): 613–616. 12096205 10.1136/thorax.57.7.613 PMC1746380

[12] C. J. Baker , V. Boulom , B. L. Reemtsen , R. C. Rollins , V. A. Starnes , and W. J. Wells , “Hemidiaphragm Plication After Repair of Congenital Heart Defects in Children: Quantitative Return of Diaphragm Function Over Time,” Journal of Thoracic and Cardiovascular Surgery 135, no. 1 (2008): 56–61. 18179919 10.1016/j.jtcvs.2007.09.031

[13] P. Bertrand and I. Sánchez , Pediatric Respiratory Diseases A Comprehensive Textbook: A Comprehensive Textbook (Springer, 2020).

[14] Nelson Textbook of Pediatrics, 20th ed. (Elsevier, 2019).

[15] M. M. ElSaegh , N. Ismail , and J. Dunning , “VATS Diaphragm Plication,” Surgical Technology International 28 (2016): 222–225. 27175808

[16] T. Watanabe , G. A. Trusler , W. G. Williams , J. F. Edmonds , J. G. Coles , and Y. Hosokawa , “Phrenic Nerve Paralysis After Pediatric Cardiac Surgery. Retrospective Study of 125 Cases,” Journal of Thoracic and Cardiovascular Surgery 94, no. 3 (1987): 383–388. 3626601

[17] J. Lemmer , B. Stiller , G. Heise , et al., “Postoperative Phrenic Nerve Palsy: Early Clinical Implications and Management,” Intensive Care Medicine 32, no. 8 (2006): 1227–1233. 16741696 10.1007/s00134-006-0208-4

[18] T. S. de Vries , B. L. Koens , and A. Vos , “Surgical Treatment of Diaphragmatic Eventration Caused by Phrenic Nerve Injury in the Newborn,” Journal of Pediatric Surgery 33, no. 4 (1998): 602–605. 9574760 10.1016/s0022-3468(98)90325-6

[19] M. de Perrot , A. Schweizer , A. Spiliopoulos , and M. Licker , “Early Improvement of Respiratory Function After Surgical Plication for Unilateral Diaphragmatic Paralysis,” European Journal of Cardio‐Thoracic Surgery 13, no. 2 (1998): 206–208. 9583830 10.1016/s1010-7940(97)00321-7

